# Chang’E-5 reveals the Moon's secrets to a longer life

**DOI:** 10.1016/j.xinn.2021.100177

**Published:** 2021-10-22

**Authors:** Ross N. Mitchell

**Affiliations:** 1 State Key Laboratory of Lithospheric Evolution, Institute of Geology and Geophysics, Chinese Academy of Sciences, Beijing 100029, China; 2 University of Chinese Academy of Sciences, Beijing 100049, China

## Main text

**Figure 1:**
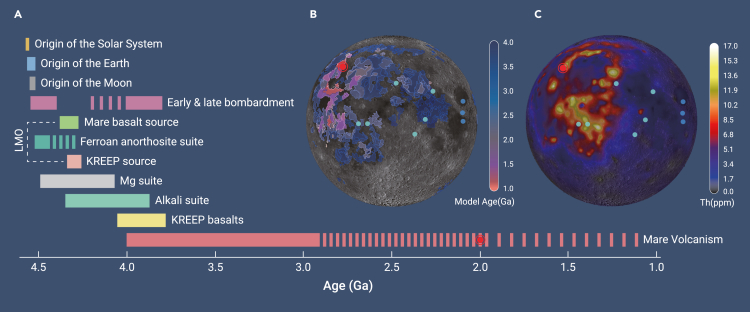
Age and location of Chang’E-5 (A) Timeline of lunar history. LMO, lunar magma ocean. Any volcanism after 2 Ga is highly localized. Cratering ages (B) and thorium contents (C) of mare basalts. In (A)–(C), red dots indicate the age and location of Chang’E-5. In (B)–(C), landing sites are indicated with dots for Apollo (light blue) and Luna (dark blue).

The Moon is not only important because it enchants us in the night sky. The Earth-Moon system is critical for Earth's surface environment and even life, with lunar gravity controlling Earth's tides and rate of rotation. The origin of the Moon, from either a giant impact or a shrinking hot debris disk, remains debated and carries implications for either early Earth or even Earth's origin, respectively. The Apollo and Luna missions provided abundant samples informing the earliest lunar history, namely the evolution of the “lunar magma ocean,” where less dense plagioclase crystallized and floated up to the surface, lending most of the lunar surface its white appearance from this anorthositic crust, but the dark patches on the Moon—its “mare” basalts, named for the large “sea” of volcanic plains—record the rest of lunar history. The youngest rocks from the Apollo and Luna missions and lunar meteorites are only dated at 2.8–2.9 billion years ago (Ga) (Figure 1A).

China's Chang’E-5 mission thus sought a new frontier: discover the younger rocks on the Moon to see what they could reveal about the longevity of lunar geologic activity. Based on crater counting chronology (see below), the selected landing site targeted potentially some of the youngest rocks on the Moon (Figure 1B). It was first critical to establish, as published in *National Science Review*,^1^ that the samples collected by Chang’E-5 from the highly impacted lunar regolith indeed had compositional affinity to mare basalts. How long did mare volcanism last, and, if volcanic activity persisted quite late in lunar history, what was the Moon's secret to a longer life? These are the questions that the Chang’E-5 samples, the first lunar samples returned to Earth in 44 years, can begin to answer. However, as with any new data, as some questions are answered, new ones always arise.

### Youngest Moon rocks

Two geochronology studies have been conducted on the Chang’E-5 basalts, both employing the Pb-Pb isochron method (^204^Pb/^206^Pb versus ^207^Pb/^206^Pb), which has proven reliable in the study of Apollo basalts. The first geochronology study of Chang’E-5 was published in *Science*^2^ on October 7^th^ and reported two types of ages: one age (1,963 ± 57 million years ago [Ma]) is based on 18 analyses of two rock fragments; the other age (2,011 ± 50 Ma) relies exclusively on Zr-bearing minerals ideal for dating due to U enrichment with negligible non-radiogenic Pb (lunar initial Pb). The second study reporting Chang’E-5 geochronology, published in *Nature*^3^ on October 19^th^, yielded a strikingly precise age of 2,030 ± 4 Ma based on 51 grains of Zr-rich minerals from 47 rock fragments. The age on Zr-rich minerals from the *Science* study^2^ agrees well with the robust 2,030 ± 4 Ma age.^3^ Zirconium-bearing minerals were only <5 μm wide, thus the ∼3-μm spot size of one study,^3^ compared with ∼7 μm in the other,^2^ also provided additional accuracy by avoiding terrestrial, laboratory-derived Pb contamination along grain boundaries. Irrespectively, the two independent laboratories in Beijing arrived at indistinguishable ages within uncertainty. The Chang’E-5 basalts are thus the youngest dated Moon rocks and they extend the duration of mare volcanism 800–900 million years longer than previously known (Figure 1A).

### Cratering chronology confirmation

The Moon is a unique resource for our being able to date the surfaces of other moons and planets in the solar system, some of which can only be dated using “crater counting” chronology. Generally, older surfaces have accumulated more impact craters and younger surfaces have less. However, translating the crater count of a rocky region into an approximate age relies heavily on the impact cratering flux in the solar system. Luckily, not only does the Moon preserve a long impacting history but it is close enough that we are able to sample rocks and precisely date them with radioisotopic dating. This allows the cratering chronology curve to be calibrated. If the impacting rate were constant, then the curve would be straight, but, based on Apollo samples, it is not, making calibration critical. Specifically, between the old and young ages of these existing samples, the cratering curve exhibits a significant change in shape, but there was a 2-billion-year-long gap in dated samples, leaving this critical portion of the curve unanchored. The 2 Ga Chang’E-5 age squarely fills this gap and anchors the cratering chronology curve.^2^^,^^3^

### A mechanism for young volcanism

Dating the youngest mare basalts thus begs the question of how volcanic activity on the Moon was sustained for so long. The small Moon is less than 30% the radius of Earth. With such a large surface-to-volume ratio (3/*r*, where *r* is the sphere's radius), the Moon should have cooled rapidly and become geologically inactive relatively early. Nonetheless, three isotopic systems (U-Pb, Sm-Nd, and Rb-Sr) all consistently indicate that the young Chang’E-5 basalts formed by the melting of the lunar mantle.^3^^,^^4^ How did the interior of the Moon keep warm for so long? On the same day as the second geochronology study,^3^ two other studies were published in *Nature*^4^^,^^5^ that investigated two possible mechanisms for how volcanic activity on the Moon lasted as late as 2 Ga.

### Is the lunar mantle wet or dry?

One possible explanation is elevated water contents in the Chang’E-5 lunar mantle source because water lowers the melting temperature, thereby requiring less heat to generate melt. Water abundances of the Moon's interior are a highly controversial topic, with estimates spanning three orders of magnitude that range between an essentially anhydrous lunar mantle to very elevated water abundances on par with Earth's mantle. Attaining the water content from the lunar mantle as calculated from the measured contents of the basaltic rocks requires backtracking the effects of magmatic evolution. The measurements of both volatile (namely, water) contents and hydrogen isotopes in both late-forming apatite minerals and ilmenite-hosted melt inclusions are together able to track the zigzagging of water contents back to the source. Following mantle melting, three magmatic stages cause water contents to increase (fractional crystallization), decrease (H_2_ degassing during melt extraction), and then increase again (crystallization of apatite). Backstripping these effects, a maximum water abundance of 1–5 mg g^−1^ is estimated for the mantle source of the Chang’E-5 basalts.^5^ Compared with Apollo basalts from ca. 4.0–2.8 Ga, the very modest water abundances of the mantle source of the Chang’E-5 basalts are among the lowest measured. Such dry mantle thus rules out any role for water in generating the Moon's youngest basalts.

### Non-KREEP origin

Another possible explanation for young lunar volcanism is an old and popular idea inspired by several lines of evidence. Within the mare basalts lies the Procellarum KREEP Terrane, where the landing site of Chang’E-5 was specifically chosen. KREEP is an acronym for a distinctive geochemical component of some lunar rocks rich in the elements K, rare-earth elements (REEs), and P. During the evolution of the lunar magma ocean, as plagioclase floated up into the crust and mafic cumulates sunk down into the mantle, incompatible elements like K and P got enriched in the residual melt, thereby potentially leaving a KREEP-rich layer in between the mantle and crust. If abundant in the upper mantle beneath Chang’E-5, radiogenic elements (U, K, and Th) may have provided sufficient heat to melt the lunar mantle at such a young age. Orbital observations indicate that Th is abundant at the surface in the region (Figure 1C), but whether the underlying mantle is also radiogenic is unknown. Furthermore, the KREEP component has never been found as a *bona fide* rock type, nor has it been tested from samples of young mare basalt. The Chang’E-5 basalts thus offered a promising test of the idea of radiogenic heating, but the results are quite unexpected. At face value, the Chang’E-5 basalts are indeed richer in incompatible elements than the Apollo and Luna basalts. However, as extensive fractional crystallization can also achieve this concentration, such an observation can arguably be a false-positive identification of the contribution of KREEP in the mantle source. Thus, isotopes resilient to magmatic evolution must be used. Both Rb-Sr and Sm-Nd isotopes indicate a depleted mantle source,^4^ in stark contrast with a KREEP affinity, which is also consistent with reported Pb isotopes.^3^ The young Chang’E-5 basalts from the KREEP Terrane have a non-KREEP origin.^4^ Thus, the prevailing notion of heat-producing elements melting the lunar mantle cannot account for young volcanic activity.

### A convection mechanism?

Chang’E-5 thus rules out two leading mechanisms for the Moon's youngest volcanism. Rejecting hypotheses is science at its most definitive, but of course the enigma of the Moon's geologic longevity now only deepens. Both the water and KREEP hypotheses are related to shallow mantle composition. Another proposed mechanism, the lunar megaregolith as a poor conductor, is an even shallower explanation. Alternatively, one might look, literally, for a deeper explanation. Mare basalts are notably only found on the nearside of the Moon. This hemispheric asymmetry may be attributed to degree 1 mantle convection (one upwelling, one downwelling), where the upwelling of core-heated mantle on the nearside caused the melting for mare volcanism. Whether the Sm-Nd isotopes or the mantle water content of Chang’E-5 at a much younger age support the continuation of a mantle convection cycle can be further investigated. Pending the discovery of supporting evidence, if lunar mantle convection was sluggish, it could have delayed the dissipation of internal heat.
